# Psychometric Properties of the Performance Enhancement Attitude Scale (PEAS) for Brazilian Sports

**DOI:** 10.3390/bs14060425

**Published:** 2024-05-21

**Authors:** Renan Codonhato, Paulo Vitor Suto Aizava, Enzo Berbery, Lenamar Fiorese

**Affiliations:** 1 Associated Post-Graduation Program in Physical Education UEM/UEL, Health Sciences Center, State University of Maringá, Maringa 87020-900, Brazil; paulo.aizava@umfg.edu.br (P.V.S.A.); lfvieira@uem.br (L.F.); 2 Department of Physical Education, Health Sciences Center, State University of Maringá, Maringa 87020-900, Brazil; ra118884@uem.br

**Keywords:** doping, performance-enhancing drugs, psychometry, sport psychology

## Abstract

Interest in psychosocial predictors of doping has been increasing as a way of finding new approaches to reduce the use of performance-enhancing drugs. This investigation aimed to test the psychometric properties of an instrument to assess doping attitudes in Brazilian athletes. The PEAS was validated in Brazilian sports through a process of translation, back-translation and content validity assessment, presenting satisfactory evidence based on its content (CVC > 0.80). Then, 994 athletes from different sexes, types of sports and competitive levels answered the Brazilian version of the PEAS. The results showed satisfactory evidence of validity based on its response process, internal structure (X^2^/df = 2.04; RMSEA = 0.032 (0.026–0.038); CFI = 0.96; TLI = 0.95) and reliability (Cronbach’s α, McDonald’s ω and CR > 0.70). Network analysis was also used to further explore the PEAS’s internal structure. Overall, the results provide support for the adoption of the PEAS for Brazilian athletes and possibly other Portuguese-speaking countries.

## 1. Introduction

Dating back to before the establishment of the World Anti-Doping Agency (WADA) in 1999, performance-enhancing drugs (PEDs) have been a major concern in sports from the lowest to the highest levels of competition [1,2]. The use of these substances, as defined and listed in the WADA code, is a direct threat to the ideal of fair competition and the overall integrity of sports while potentially risking both the physical and mental health of their users [3,4]. However, traditional anti-doping policies have been primarily based on detection and punishment, which has failed to solve the problems arising from doping in competitive sports, suggesting the need for the development and implementation of new and improved approaches [5,6].

It is understood that the factors behind the decision to dope are complex and comprise environmental, social and psychological aspects [7,8,9], thus requiring different angles of investigation and inquiry in order to better understand and deal with this phenomenon. In this sense, research on the psychosocial determinants of doping behavior in sports is growing, having the attitudes toward doping at its core [6]. Such a construct of doping attitudes has been measured and studied as a set of one’s values, views and beliefs regarding doping, and it has been considered a direct predictor of doping susceptibility and behavior [10,11].

Developed in 2002, the Performance Enhancement Attitude Scale (PEAS) [12,13] is currently the most widely adopted psychometric measurement of doping attitudes in the international sports literature [6], with higher scores representing more lenient or favorable attitudes toward doping. Such a scale has even been used in six WADA-funded research projects across Europe, Africa and Australia [14,15,16,17,18,19]. A recent systematic review and meta-analysis of the PEAS [6] found a total of 82 studies adopting this tool. Moreover, this review revealed a total of 10 different versions of the PEAS available across over 15 languages.

Despite its worldwide adoption, research using the PEAS has been most predominant in European countries (53.7%), with some contributions from Asia (22%), Oceania (8.5%), Africa (7.3%), North America (6.1%) and multinational samples (2.4%). However, no PEAS research on doping attitudes was found with South American samples [6]. Analysis of other systematic reviews of the doping psychosocial literature [10,11,20,21,22] revealed no studies from South America as well. Furthermore, manual searches performed in February 2024 for the terms “doping”, “performance-enhancing drugs” and “performance-enhancing substances” in the main South American research database (SCIELO) revealed no psychological studies of doping published within Brazilian journals, with most publications being focused on the consequences of PED use, descriptive informative data and narrative reviews. In this sense, research in South American countries can provide insights as to the validity of the PEAS in a different cultural setting.

Therefore, it is possible to observe a cultural gap in the evidence for psychosocial factors related to doping attitudes in sports, which is absent in South America, along with a limitation of the application of the PEAS in the Portuguese language. In order to fill the cultural continental gap in the doping attitude literature as well as unlock the adoption of the PEAS for Portuguese-speaking individuals, the present study seeks to translate and adapt the original 17 item version of the PEAS from English to Brazilian Portuguese and test its psychometric properties in a sample of Brazilian athletes. Present research could provide the foundations to build the capacity for and further advance both psychological and social science research on doping in Brazil and other Portuguese-speaking nations.

## 2. Materials and Methods

### 2.1. The Performance Enhancement Attitude Scale (PEAS)

The original scale in English [13] comprises 17 items for assessing athletes’ attitudes toward the use of prohibited performance-enhancing drugs and has presented strong reliability through satisfactory values for internal consistency (Cronbach’s α > 0.8), temporal stability (r = 0.75, *p* < 0.001) and CFA models (X^2^/df < 2.5). These 17 items came from an original poll of 97 items which sought to represent 26 specific topics related to performance enhancement in sports, such as hypocrisy, knowledge, known health risks, different forms of pressure, sports culture, regulations, unfair advantages, success, injuries and others. Despite the heterogeneity of items across varied subjects, the 17 items represent a unidimensional measurement of attitudes toward PEDs. Statements are answered on a 6-point Likert-type scale ranging from 1 (strongly disagree) to 6 (strongly agree) and are scored as the total sum of the items, with a possible score ranging from 17 to 102 points, where higher scores suggest a more lenient or favorable attitude toward PEDs.

### 2.2. Evidence Based on the Content of the Scale

This step followed the recommendations of Pasqualli [23]. Four experts with doctorate degrees in psychology or physical education and native Portuguese speakers fluent in the English language participated in the translation (from English to Portuguese) and back-translation (from Portuguese to English) processes. Two experts independently translated the original scale from English to Brazilian Portuguese, and these two versions were analyzed by the authors in order to produce a first version of the scale in Portuguese. The first version was then translated back into English by the remaining two experts, who worked independently.

After analyzing the agreement between each version and performing minor improvements, a new version in Portuguese was generated and presented to a small group of 20 young swimming athletes from the city of Maringá in the south of Brazil as a pilot study to obtain feedback on the response process. Suggestions were made by the athletes in order to improve comprehension. Finally, the final version of the PEAS in Brazilian Portuguese was ready for further testing, which can be referred to as the “Escala de Atitudes para Melhoria de Rendimento” (EAMR) in Portuguese.

Four other individuals, experts in the field of sports psychology, evaluated the EAMR according to its language clarity, theoretical relevance and practical pertinence. Items were rated in a scale from 1 (no clarity or relevance) to 5 (quite clear and relevant). The responses from each evaluator were averaged to compose the content validity coefficient (CVC), for which values above 0.80 were considered adequate. These experts also provided feedback and suggestions for minor adjustments.

### 2.3. Evidence Based on the Response Process, Internal Structure and Reliability of the Scale

#### Sample Characteristics

For this following step, a total of 1035 Brazilian athletes participated in the study by answering the Portuguese-PEAS with pen and paper. However, 37 participants were excluded for being under the age of 18, and other 4 responses were excluded due to incorrectly filling out the questionnaires. Thus, the final sample comprised 994 athletes with an average age of 22.79 ± 4.87 years, with 559 (56%) males and 435 (44%) females, who competed in a variety of individual (n = 495, 49.7%) and team (n = 499, 50.3%) sports. The highest competitive experience of the athletes varied from state or provincial (14.9%) to national collegiate (31.9%), national (26%) and international (27.2%) levels.

### 2.4. Procedures

The present study was approved by the Permanent Committee of Ethics in Human Research of the State University of Maringá (COPEP-UEM) under appreciation number 5.658.001. The process of gathering data took place during two different competitions: the 2018 Paraná Open Games, a state-level competition, and the 2018 Brazilian College Games, the main national collegiate level event, which were both held in the state of Paraná in the south region of Brazil. Prior to the events, authorization was obtained from the Sports Secretary of Paraná and the Brazilian Confederation of College Sports (CBDU). Athletes were approached at either the competition sites or their respective housing, according to the athlete’s availability. All participants voluntarily agreed to take part in the present research by reading and signing an informed consent form. Researchers and assistants were available to answer any questions or doubts during the questionnaires’ answering, which was performed in person with pen and paper.

### 2.5. Data Analysis

All of the following analyses were performed using MS Excel, R package and RStudio, where the packages used were readxl, xlsx, mice, psych, MVN, lavaan, qgraph, Hmisc, corrplot, polycor and semPlot. As previously described, 4 subjects were excluded from the final sample due to incorrectly filling out the questionnaire. However, a few supposedly random missing cases were still present and were kept in the dataset. These missing cases were input through the Multiple Imputation Chained Equations (MICE) algorithm. Descriptive statistics (mean, standard deviation, median, quartiles and frequency) were used for the sample characteristics and to assess items’ response patterns. Data univariate normality was tested using the Shapiro–Wilk test, which revealed a non-parametric distribution. Multivariate normality was assessed with the Mardia test, revealing a non-parametric multivariate distribution. Due to the ordinal nature of Likert scales, item-to-item correlations were calculated using polychoric correlation, while polyserial correlations were applied between the item and scale total score.

To test the internal structure of the Brazilian PEAS, confirmatory factor analysis (CFA) was conducted with the WLSMV estimation method. The following fit indices were used to assess model adequacy: adjusted chi-squared (X^2^/df < 3.00), the Tucker–Lewis index (TLI > 0.95), the comparative fit index (CFI > 0.95), root mean square error of approximation (RMSEA < 0.08) and the standardized root mean square residual (SRMR < 0.08) [24]. The average variance extracted was also assessed, for which values of AVE > 0.5 could be considered a satisfactory indicator of construct validity [25,26]. Items’ internal consistency was verified using the Cronbach’s alpha (α > 0.7), McDonald’s omega (ω > 0.7) and composite reliability (CR > 0.7) methods [27].

In line with the complexity of the doping phenomena, a last and innovative step was performed to provide another look at the scale’s internal structure: a network analysis. For this matter, Spearman correlations were performed in order to create a correlation matrix. This matrix was then used by the network and the LASSO algorithm to calculate and plot a visual network of partial correlations between variables. The least absolute shrinkage and selection operator (LASSO) forces potentially trivial and spurious correlations to zero and returns a network with only the strongest associations [28]. This method is data-driven and will provide a contrast to the theory. By producing a sparse network, we minimized the likelihood of finding false positives.

The network is composed of nodes (circles) representing variables, which are connected by lines. The color and width of such lines also reflect the direction (positive or negative) and strength (r) of the associations. Furthermore, node positioning is also calculated by the algorithm to reflect the interaction between nodes and how information travels through the network [29]. The threshold for the network was set to “TRUE”, and the regularization parameter lambda was set to a high value (λ = 0.5). To provide more information about the network and its most relevant nodes, the network centrality indices (strength, closeness and betweenness) were also calculated.

## 3. Results

### 3.1. Evidence Based on the Content of the Scale

Table 1 presents the final writing of each item in Portuguese as well as its respective original item in English, alongside the CVC for each item in Portuguese, as assessed by four independent experts. CVCi represents the items’ average score for each criterion (i.e., language clarity, theoretical relevance and practical pertinence). All of the items showed adequate scores (CVCi > 0.80), with the exception of item 10 (CVCi = 0.56), which was retained for further analysis, and despite its inclusion, the overall questionnaire score was still satisfactory (CVCtotal = 0.90). This question was also a source of doubts for some participants during data gathering, reflecting that its content might need to be better adapted to the Portuguese language in order to convey the same original meaning with clarity.

### 3.2. Evidence Based on the Response Process

**Figure 1:**
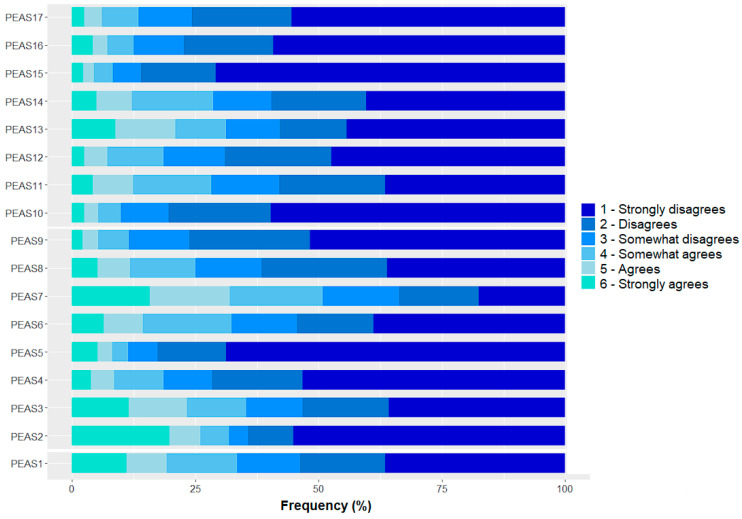
Frequency of item responses for PEAS of Brazilian athletes.

The frequency of item responses (Figure 1) showed a clear predominance of scores ranging between 1 (strongly disagree) and 3 (somewhat disagree), meaning that the majority of the sample disagreed with the statements being presented and thus had negative attitudes toward doping. In this sense, it is possible to highlight items 5 (“Athletes should not feel guilty about breaking the rules and taking performance enhancing drugs.”) and 15 (“Doping is not cheating since everyone does it.”) as the most disagreed upon items. In simpler terms, the present sample mostly agreed that athletes should feel guilty for breaking the rules and that doping is in fact cheating, whether or not others opt to do it, which suggests the involvement of morals or sports ethics as factors that could keep these athletes away from doping.

On the other hand, item 7 (“Health problems related to rigorous training and injuries are just as bad as from doping.”) divided the sample, with about 50% agreeing or disagreeing to some degree with this statement. Such a result might suggest a possible entry for athletes who are already sacrificing some of their health in exchange for performance and are willing to go further or those who are looking for compensation due to the time lost while injured. Item 2 presented the higher frequency of extreme responses, with almost a quarter of the sample strongly agreeing that “Doping is necessary to be competitive”.

### 3.3. Evidence Based on the Internal Structure and Reliability of the Scale

Diving further into the scale’s characteristics, Table 2 presents the correlation matrix of the items and the single dimension of doping attitudes. The majority of the item-item correlations ranged between r = 0.15 and 0.50, which is desirable in order to avoid possibly redundant items (r > 0.50) or items that do not share their variance with the remaining items within the same dimension or factor (r < 0.15). Item 6 (“Athletes are pressured to take performance-enhancing drugs.”) was the most independent item, sharing mostly weak item-item correlations and the smallest item-total correlation. Meanwhile, item 15 (“Doping is not cheating since everyone does it.”) presented the highest item-total correlation (r = 0.79), followed by items 4 (recreational drugs) and 8 (media exaggeration) (r = 0.71).

For the next step of the analysis, a CFA model was tested with direct paths between a single latent factor representing doping attitudes and each of the 17 items (Model 1). Overall, this first model did not perform poorly. However, an acceptable fit was not achieved due to high adjusted chi-squared (>3.00) and low CFI and TLI values (<0.95) (X^2^/df = 4.54; *p* <0.01; RMSEA = 0.06 (0.055–0.065); p-RMSEA < 0.01; CFI = 0.84; TLI = 0.82; SRMR = 0.057).

**Figure 2:**
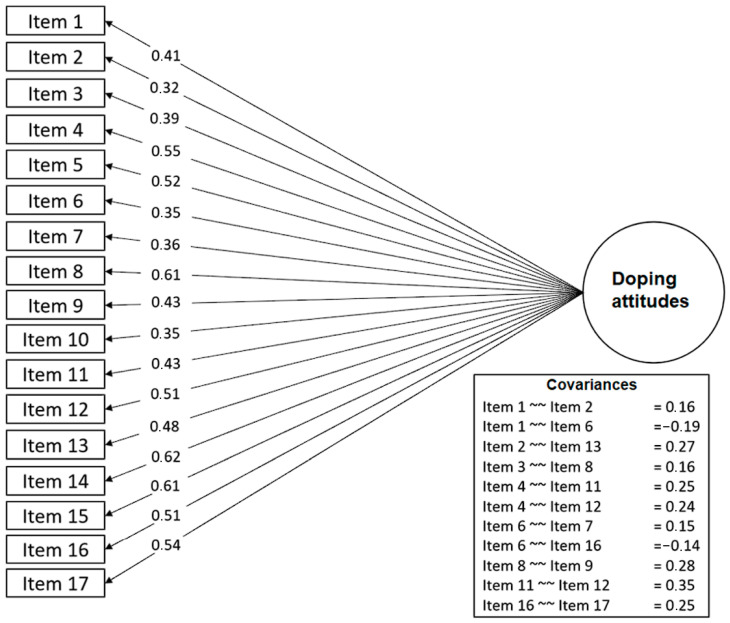
Factorial structure for the PEAS in Brazilian athletes. Note: All paths and covariances were statistically significant (*p* < 0.01).

In order to achieve the desired model fit indices, further steps of analysis were conducted by adding covariances between the items according to the suggested modification indices, which would be summed up in the final model. A total of 11 covariances were added, for instance, between statements on recreational drugs (items 4, 10, 11 and 12), media opinion (items 8 and 9), the need or unavoidability of doping (items 2 and 3) and underestimation of doping-related health problems (items 3 and 8). The final model (Figure 2) presented an acceptable fit and good evidence of construct validity (X^2^/df = 2.04; *p* < 0.01; RMSEA = 0.032 (0.026–0.038); p-RMSEA = 1.00; CFI = 0.96; TLI = 0.95; SRMR = 0.035).

We opted for the route of allowing covariances instead of excluding items because a few factors suggested that each item had its contribution to the overall factor. Since the factor loadings were all close to or greater than 0.40, the standardized residuals were low (<2) (Mullan et al., 1997 [30]), the expected change was >0.25 according to the modification indices, and all items retained the factor loadings’ significant *p* values (z-score > 1.96) (Brown, 2015 [31]). Aside from this, previous analysis (Table 2) did not point toward strong collinearity for the items. Since there is still no consensus regarding a single short-version for this scale [6], preserving the items and their respective information seemed reasonable for the time being, raising the need for further investigation to determine if there is a consensus for a short form of the PEAS in Brazilian Portuguese.

The reliability indicators were all satisfactory (>0.80) according to Cronbach’s alpha (α = 0.83 (0.81–0.84)), McDonald’s omega (ω = 0.84) and composite reliability (CR = 0.83). However, only 23% of the average variance of the scale was explained (AVE = 0.23), which falls below the desired value of 0.50. A possible explanation for this result might be the complex and multifactorial nature of the investigated phenomenon, for which single items will hardly ever be able to explain high amounts of variance, attesting to the difficulty of trying to estimate and predict one’s attitudes toward doping.

**Figure 3:**
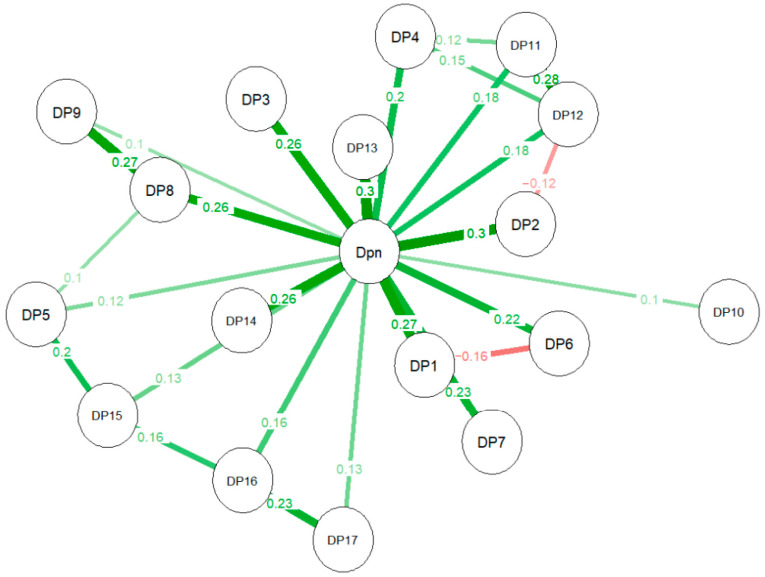
LASSO network of items and doping attitudes of Brazilian athletes. Note: Red lines represent indirect relationships.

The network analysis results (Figure 3) supported the findings from the CFA model. The total score of the PEAS was positioned at the center of the network, with all items distributed across all directions with no significant clustering of items. This structure also shows that all items had a direct connection to the main construct of doping attitudes, and despite a series of associations between items, no second structure or dimension emerged, reinforcing the scale’s unidimensional characteristic. Similar to the CFA model, items interacted with each other but were still better explained by the single latent factor of doping attitudes. Another similarity was the low strength of associations (r ≤ 0.30), as evidenced through the AVE result.

**Figure 4:**
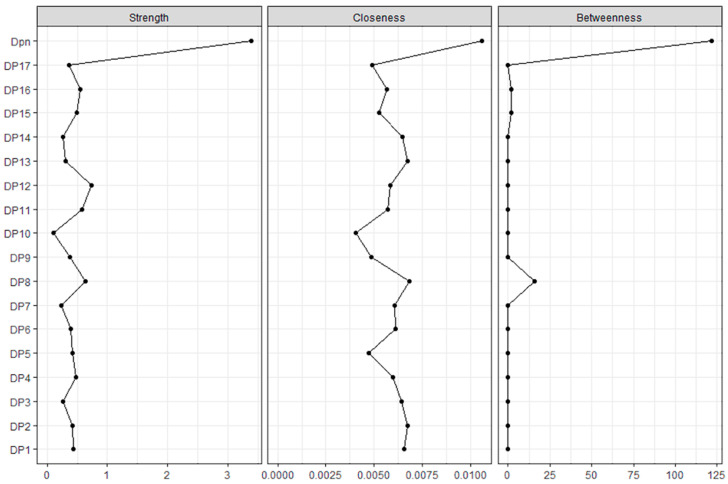
Network centrality indices.

Network centrality indices (Figure 4) aid in finding the most influential or important nodes within a complex network. Items 8, 11 and 12 presented the higher degrees of strength of associations. However, only item 8 acted as a bridge connecting a few other nodes. Moreover, all other nodes had zero degrees of betweenness, reinforcing their single and direct connection to the main construct of doping attitudes. The levels of closeness were low overall and similar between nodes. Due to its levels of closeness and betweenness being among the best and being the only item node with some degree of betweenness, the results suggest that item 8 is the most influential node in the network, apart from the total score of the doping attitudes.

### 3.4. Measurement Invariance

Based on the final CFA model (Figure 2), two other series of CFA were performed in order to test the measurement invariance between males and females (Invariance Model 1: sex) and between athletes from individual and team sports (Invariance Model 2: sport type). The compound results of all invariance models are shown in Table 3. The PEAS reached the highest degree of model invariance (full uniqueness) for both sex and sport type, meaning that the scale’s configural structure, loadings, intercepts and residuals did not vary according to the athletes’ sex or type of sport.

## 4. Discussion

The present investigation aimed to perform a transcultural adaptation of the PEAS to the context of Brazilian sports, providing evidence of validity based on the content, reliability and internal structure of the scale. Despite being the most widely adopted tool to assess doping attitudes in psychological and social research and already being available in many languages, this was the first work to translate and adapt the PEAS to Portuguese. In this sense, the present study fills a major gap in psychological doping inquiry by making this important psychometric tool available to Brazilian and other Portuguese-speaking researchers and those who work with sports, aside from being the first study to bring evidence from the PEAS in a South American sample.

Overall, the scale presented satisfactory indices in all steps of the process. The committee of experts found that the items in the Brazilian Portuguese version of the scale had clear language, theoretical relevance and practical pertinence (CVC = 0.90). Correlations between items and items and dimensions were, for the most part, low to moderate (r = 0.15 to 0.50), which suggests that the items were somewhat related but with no significant levels of collinearity or existence of a second factor, possibly highlighting the unique contribution of each statement in the scale. The final CFA model, allowing for 11 covariances, presented an excellent fit (X^2^/df = 2.04; *p* < 0.01; RMSEA = 0.032 (0.026–0.038); p-RMSEA = 1.00; CFI = 0.96; TLI = 0.95; SRMR = 0.035). All three reliability indicators showed satisfactory values as well (α, ω and CR > 0.8). The network analysis provided further support for the PEAS’s internal consistency and reliability by having a data-driven multivariate representation of the scale’s item dynamics.

This was the first study to apply network analysis to study the PEAS’s content and structure. Such a method is still not common in psychometric or even exploratory research in sports sciences. However, it is a powerful tool for analyzing complex relationships and patterns, visualizing data and revealing the core features of a complex system which can offer great application for psychological inquiry [32]. Interestingly, the results from the network analysis provided support for the factor structure found through CFA that added covariances instead of excluding items, as nine item-item associations emerged on a network with a single factor, where each item was directly and mainly connected to the overall attitude dimension, showing items’ uniqueness and individual contributions, as well as a high degree of covariance between items.

In the present study, we opted to maximize item retention in the CFA model due to the sufficient strength of the factor loadings (close to or above 0.40), low standardized residuals (<2), sufficient expected change according to the modification indices (>0.25) and factor loadings’ significant *p* values (z-score > 1.96) [30,31]. This approach was also observed in the French validation study [33], which also included a high number of covariances—eight in total—as opposed to excluding items.

While the Spanish version of the scale also maintained all 17 items, adding only 3 covariances to the larger sample, it is important to mention that the authors in this study only used the normalized chi-squared and RMSEA fit indices to state that the scale had an adequate fit without the need for further inclusion of covariances [34], while the CFI and TLI, the main reason for the model adjustments in the present study, were not presented. Moreover, Persian versions of the scale were found with 9 [35] and 14 items [36]. The German version of the PEAS by Elbe and Brand [37] included only 6 items, and the Polish version had 11 items [38].

It is possible to observe a tendency to exclude PEAS items and aim for shorter versions. The number of items in the PEAS is still a subject of debate, as no consensus has been found between authors. The systematic review of the PEAS by Folkerts et al. [6] revealed 10 different and shorter versions of the scale, with no single item from the original scale being present in any other versions. These authors even suggest that a revised short version of the scale is needed for a “master version” to be used consistently across investigations. Based on the present findings and the psychometric literature for the PEAS, we suggest that original studies should always start with the complete 17 item version. Nevertheless, the complete version with all 17 items is still the most adopted one [6], which can then be refined through further data analysis.

Considering the complexity of doping as a whole, which encompasses an immeasurable interaction between individual and sociocultural factors which can play a role in an athlete’s decision to dope or stay clean, it is not too difficult to understand why so much contrast is present in the literature. Such complex factors might also be responsible for the lower-than-ideal values for the AVE reported in the present findings. Moreover, it is possible that this is the first study to even report the values of the AVE for this scale.

Despite all of the controversy present in the literature, our findings are enough to support and recommend the adoption of the Brazilian Portuguese version of the PEAS proposed here. The majority of the problems and inconsistencies found across the wide range of PEAS versions might reflect the complex nature of the doping phenomena itself rather than major problems with the scale and its statements. The present work offers important contributions to both the ever-growing literature on the PEAS itself and the field of sport psychology in Brazil, which is rather limited in terms of the number of validated research tools. The use of network analysis might improve the debate on the number and relevance of items by presenting a visualization of how items behave within the scale or relate to other variables of interest. Making the PEAS available in Portuguese is a major contribution to the literature on doping attitudes, as it offers evidence from new cultural settings to the literature as whole, as well as helping local researchers, sport psychologists, coaches and athletes be able to better understand and work with this phenomenon based on the national literature, as evidence from other cultures might not apply accordingly when working with, for example, Brazilian athletes.

Still, some limitations are worth mentioning. By seeking to validate an instrument for the overall Brazilian population, a heterogenous sample was adopted and included individuals from all regions of the country across a variety of sports, which limited our understanding of the specificities related to doping attitudes. However, the measurement invariance models suggest that the goal of producing a scale for the broader population of Brazilian athletes might have been achieved, at least in terms of sex and type of sport. Since this is the first study to analyze the psychometric properties of the PEAS for this language, comparisons to further compare and discuss the adoption, exclusion or rewriting of items were also limited. Another limitation was the assessment of criterion-related validity. Due to the nature of the outcome of interest (i.e., doping) being illegal and taboo among many athletes in competitive settings, the authors chose to not include a direct assessment of doping, such as self-reported use or intentions, to not discourage athletes’ participation, as the data were gathered by pen and paper during competitions.

Future investigations should carry on the continuous process of scale validation and refinement by providing more evidence of validity across other sports populations in Brazil as well as other Portuguese-speaking countries. Further analysis should include correlation variables in order to assess external validity and provide psychological evidence of doping attitudes in Brazil, since there is an absolute absence of studies on this specific topic in the entirety of South American journals. Moreover, future studies should attempt to test the criterion validity of the PEAS in Portuguese-speaking athletes by adding more direct estimates of doping behavior, such as self-reported doping use or intentions to dope.

## 5. Conclusions

In order to summarize our findings, it is possible to conclude that the Portuguese version of the PEAS is a reliable and valid tool for assessing doping attitudes in the context of Brazilian sports. All indicators presented satisfactory values, and the measurement invariance model reached full uniqueness, showing that the construct at hand was consistent across samples in terms of both sex and type of sport (team and individual). Despite the inconsistencies found in the literature regarding the number of items to be retained, it was possible to conclude that each item offered a unique contribution and a different aspect related to doping attitudes, as shown by the network analysis. As highlighted through items 5 and 15, the involvement of morals and sports ethics might be an important factor that could keep athletes away from doping. Meanwhile, professionals should be aware of training-related injuries and health problems, as item 7 was the most agreed upon item and could represent a factor raising the athletes’ interest in doping as a way to compensate for such training-related factors. Lastly, we hope that researchers and professionals working with doping through the lens of sport psychology will benefit from the present tool now being available in a new language, which could offer new cultural insights.

## Figures and Tables

**Table 1:** CVC scores for the translated items of the PEAS in Brazilian Portuguese.

	Item	CVCi
1	Legalizar produtos para melhoria de rendimento seria benéfico para o esporte.	0.93
2	Doping é necessário para ser competitivo.	0.93
3	Os riscos relacionados ao doping são exagerados.	0.99
4	Drogas recreativas motivam para treinar e competir em alto nível.	0.96
5	Atletas não deveriam se sentir culpados por quebrar as regras e usar drogas para melhorar o rendimento.	0.96
6	Atletas são pressionados a usar drogas para melhorar o rendimento.	0.96
7	Problemas de saúde relacionados ao treinamento rigoroso e às lesões são tão ruins quanto o doping.	0.93
8	A mídia trata a questão do doping de forma desproporcional.	0.83
9	A mídia deveria falar menos sobre o doping.	0.99
10	Atletas não têm nenhuma opção de escolha de carreira, a não ser o esporte.	0.56
11	Atletas que usam drogas recreativas, as usam porque ajudam em situações esportivas.	0.93
12	Drogas recreativas ajudam a superar o tédio durante os treinamentos.	0.93
13	Doping é uma parte inevitável do esporte competitivo.	0.96
14	Atletas muitas vezes perdem tempo devido a lesões, e as drogas podem ajudar a compensar esse tempo perdido.	0.99
15	Doping não é trapaça, já que todo mundo faz uso.	0.93
16	Somente a qualidade do rendimento deveria importar, não a forma como os atletas o alcançam.	0.93
17	Não há diferença entre drogas e o equipamento técnico que pode ser usado para melhorar o rendimento (ex.: câmara hipobárica para simular altitude, maiôs velozes, varas de fibras de vidro).	0.86
	CVC total	0.90

**Table 2:** Polychoric and polyserial correlations between items and PEAS total scores of Brazilian athletes (n = 994).

	1	2	3	4	5	6	7	8	9	10	11	12	13	14	15	16	17
Item 1	-																
Item 2	0.34	-															
Item 3	0.30	0.20	-														
Item 4	0.36	0.36	0.34	-													
Item 5	0.33	0.25	0.31	0.47	-												
Item 6	−0.01	0.10	0.11	0.23	0.21	-											
Item 7	0.14	0.18	0.19	0.23	0.25	0.29	-										
Item 8	0.33	0.24	0.43	0.43	0.54	0.29	0.30	-									
Item 9	0.26	0.24	0.27	0.30	0.40	0.16	0.22	0.58	-								
Item 10	0.16	0.10	0.19	0.26	0.29	0.17	0.19	0.23	0.35	-							
Item 11	0.19	0.20	0.21	0.52	0.30	0.26	0.20	0.35	0.26	0.26	-						
Item 12	0.22	0.15	0.20	0.56	0.37	0.29	0.25	0.42	0.32	0.28	0.59	-					
Item 13	0.22	0.47	0.23	0.34	0.30	0.27	0.24	0.33	0.28	0.23	0.27	0.37	-				
Item 14	0.25	0.26	0.33	0.39	0.40	0.35	0.30	0.45	0.31	0.25	0.39	0.46	0.46	-			
Item 15	0.34	0.31	0.31	0.43	0.60	0.25	0.22	0.53	0.47	0.38	0.34	0.43	0.46	0.49	-		
Item 16	0.35	0.23	0.29	0.35	0.47	0.11	0.20	0.41	0.41	0.33	0.26	0.33	0.34	0.45	0.59	-	
Item 17	0.22	0.16	0.25	0.35	0.43	0.25	0.25	0.40	0.35	0.33	0.32	0.42	0.35	0.49	0.54	0.59	-
Total Score	0.51	0.54	0.52	0.71	0.70	0.43	0.47	0.71	0.56	0.48	0.57	0.64	0.62	0.68	0.79	0.64	0.65

**Table 3:** PEAS measurement invariance models for a sample of Brazilian athletes (n = 994).

Model	X^2^/df	P	RMSEA	p-RMSEA	CFI	TLI	SRMR
Sex							
1: Configural	1.42	0.00	0.026	1.00	0.988	0.984	0.039
2: Metric	1.31	0.00	0.027	1.00	0.985	0.983	0.044
3: Scalar	1.29	0.00	0.026	1.00	0.985	0.984	0.045
4: Full uniqueness	1.31	0.00	0.028	1.00	0.983	0.982	0.051
Type of sport							
1: Configural	1.46	0.00	0.027	1.00	0.987	0.984	0.039
2: Metric	1.24	0.00	0.024	1.00	0.989	0.987	0.042
3: Scalar	1.51	0.00	0.034	1.00	0.976	0.973	0.048
4: Full uniqueness	1.58	0.00	0.037	1.00	0.969	0.968	0.055

## Data Availability

Data are contained within the article.
